# Post-transplant Inflammatory Bowel Disease Associated with Donor-Derived TIM-3 Deficiency

**DOI:** 10.1007/s10875-024-01667-z

**Published:** 2024-02-16

**Authors:** Adrian Baldrich, Dominic Althaus, Thomas Menter, Julia R. Hirsiger, Julius Köppen, Robin Hupfer, Darius Juskevicius, Martina Konantz, Angela Bosch, Beatrice Drexler, Sabine Gerull, Adhideb Ghosh, Benedikt J. Meyer, Annaise Jauch, Katia Pini, Fabio Poletti, Caroline M. Berkemeier, Ingmar Heijnen, Isabelle Panne, Claudia Cavelti-Weder, Jan Hendrik Niess, Karen Dixon, Thomas Daikeler, Karin Hartmann, Christoph Hess, Jörg Halter, Jakob Passweg, Alexander A. Navarini, Hiroyuki Yamamoto, Christoph T. Berger, Mike Recher, Petr Hruz

**Affiliations:** 1 Immunodeficiency Laboratory, Department of Biomedicine, University of Basel, Basel, Switzerland; 2 Gastroenterology and Hepatology, University Center for Gastrointestinal and Liver Diseases, Clarunis, Basel, Switzerland; 3 Pathology, Institute of Medical Genetics and Pathology, University Hospital Basel, University of Basel, Basel, Switzerland; 4 Translational Immunology, Department of Biomedicine, University Hospital, Basel, Switzerland; 5 Molecular Diagnostics, Laboratory Medicine, University Hospital Basel, Basel, Switzerland; 6 Allergy and Immunity Laboratory, Department of Biomedicine, University Hospital Basel and University of Basel, Basel, Switzerland; 7 Translational Diabetes, Department of Biomedicine, University Hospital, Basel, Switzerland; 8 Division of Hematology, University Hospital Basel, Basel, Switzerland; 9 Department of Oncology and Hematology, Kantonsspital Aarau, Aarau, Switzerland; 10 Competence Center for Personalized Medicine, University of Zürich/Eidgenössische Technische Hochschule (ETH), Zurich, Switzerland; 11 Division Medical Immunology, Laboratory Medicine, University Hospital Basel, Basel, Switzerland; 12 Department of Endocrinology, Diabetology and Clinical Nutrition, University Hospital Zurich (USZ) and University of Zurich (UZH), Zurich, Switzerland; 13 Cancer Immunology, Department of Biomedicine, University Hospital Basel and University of Basel, Basel, Switzerland; 14 Department of Rheumatology, University Hospital Basel, Basel, Switzerland; 15 Division of Allergy, Department of Dermatology, University Hospital Basel and University of Basel, Basel, Switzerland; 16 Immunobiology Laboratory, Department of Biomedicine, University Basel Hospital, Basel, Switzerland; 17 Department of Medicine, Cambridge Institute of Therapeutic Immunology & Infectious Disease, University of Cambridge, Cambridge, UK; 18 Dermatology, University Hospital Basel, Basel, Switzerland; 19 Research Group 2, AIDS Research Center, National Institute of Infectious Diseases, Tokyo, Japan; 20 University Center for Immunology, University Hospital Basel, Petersgraben 4, 4031 Basel, Switzerland

**Keywords:** Inborn errors of immunity, inflammatory bowel disease, *HAVCR2*, TIM-3, immune checkpoint, stem cell transplantation

## Abstract

Inflammatory bowel disease (IBD) occurring following allogeneic stem cell transplantation (aSCT) is a very rare condition. The underlying pathogenesis needs to be better defined. There is currently no systematic effort to exclude loss- or gain-of-function mutations in immune-related genes in stem cell donors. This is despite the fact that more than 100 inborn errors of immunity may cause or contribute to IBD. We have molecularly characterized a patient who developed fulminant inflammatory bowel disease following aSCT with stable 100% donor-derived hematopoiesis. A pathogenic c.A291G; p.I97M *HAVCR2* mutation encoding the immune checkpoint protein TIM-3 was identified in the patient’s blood-derived DNA, while being absent in DNA derived from the skin. TIM-3 expression was much decreased in the patient’s serum, and in vitro-activated patient-derived T cells expressed reduced TIM-3 levels. In contrast, T cell-intrinsic CD25 expression and production of inflammatory cytokines were preserved. TIM-3 expression was barely detectable in the immune cells of the patient’s intestinal mucosa, while being detected unambiguously in the inflamed and non-inflamed colon from unrelated individuals. In conclusion, we report the first case of acquired, “transplanted” insufficiency of the regulatory TIM-3 checkpoint linked to post-aSCT IBD.

**Supplementary Information:**

The online version contains supplementary material available at 10.1007/s10875-024-01667-z.

## Introduction

Inborn errors of immunity (IEI) are a large group of genetically determined diseases caused by germline loss- or gain-of-function mutations in immune-related genes. More than 480 IEI entities have been described to date [1]. More than 100 of those can be associated with IBD [2, 3]. The clinical penetrance of IEI-related mutations is often low to moderate [4]. However, immune-dysregulating post-transplant events, such as lymphopenia, treatment with immune-regulating drugs or infections might increase the clinical penetrance [4, 5]. In addition, environmental factors, such as the herbizide propyzamide, contribute to IBD pathogenesis [6]. The clinical phenotype of IEI is often broad and may even vary within carriers of the exact same variant in a family [7, 8].

The occurrence of inflammatory bowel disease (IBD) in non-IEI patients after allogeneic stem cell transplantation for hematological malignancies is extremely rare [9–13]. No molecular explanation has yet been uncovered in these rare cases. Allogeneic stem cell transplantation (aSCT) can be curative for patients with hematological malignancies and even represents a potentially beneficial treatment option for severe treatment-refractory Crohn’s disease [14]. Post aSCT, patients can develop graft-versus-host disease (GvHD) affecting various organs, including the gut, which is caused by donor-derived T cell-mediated inflammation [15]. Histological findings of GvHD of the small bowel and colon are characterized by crypt epithelial cell apoptosis and segmental crypt loss or fibrosis of the submucosa, with or without a dense lymphocytic infiltrate [16]. By contrast, the pathogenesis of a post-aSCT IBD remains unclear. Since aSCT donors are currently not genetically screened for carriage of rare IEI variants, there is a non-neglectable risk of transferring risk alleles potentially modifying post-transplant immune health in general and post-aSCT IBD in particular.

## Methods

### Consent

The prospective cohort study of the functional and genetic architecture of patients with primary immune dysregulation (FuGe-PID) has been approved by the ethics commission of Northwestern Switzerland (EKNZ215-187).

### Flow Cytometric Analysis of T Cells and In Vitro Stimulation of Peripheral Blood Mononuclear Cells (PBMC)

Lymphocyte flow cytometric phenotyping of peripheral blood depicted in Supplemental Table 1 was performed as part of the routine immunology laboratory diagnostics of the Basel University Hospital as described [17]. Peripheral blood mononuclear cells (PBMCs) were isolated by standard density-gradient centrifugation (Lymphoprep, Fresenius Kabi). For detection of TIM-3 expression, freshly isolated PBMCs were adjusted to a concentration of 2 × 10^6^/ml and stimulated with 5 µg/ml phytohemagglutinin (Sigma-Aldrich) and 0.325 IE/ml IL-2 (Novartis). Immune cell populations and Tim-3 expression were identified by staining with fluorophore-conjugated anti-CD4, anti-CD8, anti-CD25, and anti-Tim-3 vs. mouse IgG1 κ isotype control (all BioLegend) at 4 °C for 30 min. For detection of cytokine production, flat bottom plates were coated overnight at 4 °C with 1 µg/ml agonistic anti-CD3 (OKT3, BioLegend) and 2 µg/ml agonistic anti-CD28 (CD28.2, BioLegend). Freshly isolated PBMCs were adjusted to 2 × 10^6^/ml and incubated for 4 h on the previously coated plate in the presence of Brefeldin A (BioLegend). Cytokine expression was identified by staining with fluorophore-conjugated anti-CD3, anti-CD4, anti-CD8, anti-IFNG, anti-IL17, and anti-CD69 (all BioLegend). Fluorescence data was acquired using CytoFLEX (Beckman Coulter) cytometers and BD LSR Fortessa (BD Biosciences). Cell populations and median fluorescence intensities (MFI) were determined using FlowJo (BD Biosciences, formerly developed by FlowJo LLC). In vitro stimulated PBMC as described above were additionally cultured in the absence of Brefeldin A, and 4 h later, cell pellets were stored in RNA lysis buffer to analyze cytokine mRNA production. A full description of the used antibodies is provided in Supplemental Methods.

**Table 1:** Lymphocyte and T cell subpopulations of the patient in clinical remission (under infliximab therapy) compared to validated internal reference values

	Normal range	Patient (year: 2021)
T cell relative	55–86% (of lymphocytes)	66%
T cell absolute	742–2750/μl	751/μl
B cell relative	5–22% (of lymphocytes)	27%
B cell absolute	80–616/μl	309/μl
NK cell relative	5–26% (of lymphocytes)	6%
NK cell absolute	84–724/μl	67/μl
CD4 T cell relative	33–58% (of lymphocytes)	44%
CD4 T cell absolute	404–1612/μl	488/μl
CD8 T cell relative	13–39% (of lymphocytes)	21%
CD8 T cell absolute	220–1129/μl	233/μl
Naive CD4 T cells	15.7–54.7% (of CD4)	11.7%
Central memory CD4 T cells	8–28.9% (of CD4)	45.6%
Effector memory CD4 T cells	16.8–57.4% (of CD4)	41.2%
Terminal differentiated CD4 T cells (TEMRA)	3.6–23.2% (of CD4)	1.4%
Recent thymic emigrant CD4 T cells	14.1–37.2% (of CD4)	11.2%
Follicular CD4 T cells	6.9–19.1% (of CD4)	26.9%
Regulatory CD4 T cells	6.1–11.0% (of CD4)	10.2%
Activated CD4 T cells	4.1–15.6% (of CD4)	12.0%
Naïve CD8 T cells	7.0–62.5% (of CD8)	13.2%
Central memory CD8 T cells	0.6–4.4% (of CD8)	10.0%
Effector memory CD8 T cells	4.3–64.5% (of CD8)	61.7%
Terminal differentiated CD8 T cells	8.1–60.5% (of CD8)	15.1%
Activated CD8 T cells	8.7–45.2% (of CD8)	24.9%

### Flowcytometric Assessment of Monocytes

1.5 × 10^6^ PBMC were stained for 30 min at 4 °C using antibodies against CD163 (BV421, clone GHI/61), CCR2 (BV510, clone K036C2), CD14 (BV605, clone MSE2), CD16 (APC-Fire750, clone 3G8), CD11b (BV711, clone ICRF44), HLA-DR (A488, clone L243), CD45 (PerCpCy5.5, clone HI30), CD209 (PE, clone 9E9A8), CD19 (PECy5, clone HIB19), CD3 (PECy5, clone UCHT1), CD56 (PECy5, clone 5.1H11), CD20 (PECy5, clone 2H7), TCRb (PECy5, clone UP26), and Fc block (clone FC1). All antibodies were purchased from BioLegend, except for Fc block which was obtained from BD Biosciences. Cell analysis was performed on a FACS LSRII Fortessa (BD Biosciences). Acquired data were analyzed using FlowJo software (Version 10.8.1, BD Biosciences).

### RNA Isolation, cDNA Generation, and Real-Time PCR Analysis

RNA isolation was performed using a QIAamp RNA blood mini kit according to the manufacturer’s protocol (Qiagen). Before reverse transcription, RNA was digested with DNAse I Amplification Grade Kit (Sigma) at 37 °C for 30 min. Afterwards, DNAse was inactivated at 65 °C for 10 min. Next, random primers (Promega) were annealed at 70 °C for 5 min, and cDNA was synthesized by GoTaq G2 DNA Polymerase (Promega) in a TProfessional TRIO PCR Thermocycler (Core Life Sciences) according to the manufacturer’s protocol. cDNA levels were assessed by quantitative PCR in a qPCR cycler ABI machine (Applied Biosystems, Thermo Fisher Scientific) using a Sybr Green method according to the manufacturer’s protocol (Promega). As reference genes for normalization, we used 18 s rRNA, Actin, and IPO8. For every gene, the mean expression of all control samples was set to 100%, and each individual sample was normalized to this (% of control).

### Whole Exome Sequencing and Targeted Sanger Sequencing

Whole exome sequencing and bioinformatics were performed as recently described [17].

Peripheral blood or skin-derived genomic DNA of the index patient was isolated using the QIAamp® DNA Mini kit (Qiagen) according to the manufacturer’s protocol. DNA was amplified by polymerase chain reaction (PCR). All primers used were designed by Primer-BLAST and are listed in Supplemental Methods.

End-point PCR was performed using the GoTaq G2 DNA Polymerase (Promega) according to the manufacturer’s protocol with a primer concentration of 0.5 µM. PCR was performed on a TProfessional TRIO PCR Thermocycler (Core Life Sciences). PCR products were separated and visualized on a 1.5% agarose gel. Specific bands were cut and purified using the Qiagen Gel Extraction kit. Sanger sequencing was performed by the company Microsynth.

To generate complementary DNA (cDNA) of the index patient, RNA was isolated using the QIAamp® RNA Blood Mini Kit (QIAGEN) according to the manufacturer’s protocol. RNA concentration was determined using the NanoDrop 2000c (Thermo Fischer Scientific). DNA was digested with the DNase I Amplification Grade Kit (Sigma-Aldrich). Random primers (Promega) were annealed at 70 °C for 5 min. cDNA synthesis was performed according to the Qiagen’s GoScript Reverse Transcription System protocol in a TProfessional TRIO PCR Thermocycler (Core Life Sciences).

### Real-Time PCR Analysis of Inflammatory Cytokine Expression

RNA isolation was performed using a QIAamp RNA blood mini kit according to the manufacturer’s protocol (Qiagen). Prior to reverse transcription, RNA was digested with DNAse I Amplification Grade Kit (Sigma) at 37 °C for 30 min. Afterwards, DNAse was inactivated at 65 °C for 10 min. Next, random primers (Promega) were annealed at 70 °C for 5 min, and cDNA was synthesized by GoTaq G2 DNA Polymerase (Promega) in a TProfessional TRIO PCR Thermocycler (Core Life Sciences) according to the manufacturer’s protocol. The freshly synthesized cDNA was examined by quantitative PCR in a qPCR cycler ABI machine (Applied Biosystems, Thermo Fischer Scientific) using a Sybr Green method according to the Manufacturer’s protocol (Promega). As reference genes for normalization, we used *18 s rRNA*, *ACTB* (actin), and *IPO8*. Afterwards, we calculated the mean of all controls and applied it to each individual sample to calculate the percent change compared to the norm. Primers used are listed in Supplemental Methods.

### Pathology Studies and Immunohistology

A TIM-3 specific monoclonal antibody (clone D5D5R, Cell Signaling, Danvers, MA, USA) was used to detect TIM-3 in immune histology. A CD5-specific monoclonal antibody (clone SP19, Ventana/Roche, Tucson, AZ, USA) was used to stain mucosal T cells in general. FOXP3 was stained with an antibody from ABCAM (clone SP97), and ROR-γt was stained with an antibody from Biocare Medical (clone 6F3.1).

### Quantification of Serum TIM-3 and Galectin-9 Levels by ELISA

Serum TIM-3 and Galectin-9 levels were determined by ELISA kits as recently reported [18] in multiple sera from the index patient (patient) over a period of 6 years (2017–2023), sera from healthy controls (control), and sera from randomly assigned patients enrolled into our prospective immune-dysregulation cohort (disease control).

### Chimerism Analysis in Sorted Lymphocyte and Monocyte Populations

For chimerism analysis, we took advantage of the sex mismatch between patient (male) and donor (female) and used a diagnostically validated, highly sensitive (limit of detection, LoD 0.01%) digital PCR-based approach to specifically amplify the *SRY* gene sequence on the Y chromosome. CD3^+^CD4^+^ vs. CD3^+^CD8^+^ T cells and CD56^+^CD3^−^ NK cells of the index patient were flow cytometrically sorted, and monocytes were enriched by using a monocyte rosette separation kit (STEMCELL Technologies).

## Results

A currently 50-year-old male patient was treated with a 10/10 HLA allele-matched, unrelated, and sex-mismatched (female donor) allogeneic stem cell transplantation (aSCT) in August 2012 due to a T cell prolymphocytic leukemia. The clinical status of the stem cell donor was requested from the bone marrow registry, and well-being had been documented at the last 5 years after the transplant. A conditioning regimen with the BEAM-Fludarabine 2Gy total body irradiation protocol (carmustine, etoposide, cytarabine, melphalan, and fludarabin) was applied in combination with anti-thymocyte globulin (ATG) to reduce the recurrence of malignant T cells. The post-transplant course was complicated by acute graft-versus-host disease (GvHD, overall stage 3) of the skin (stage 3), liver (stage 2), and intestine (stage 1) occurring 2 weeks after aSCT. Acute GvHD which was well-controlled by steroids (which were subsequently tapered) and single administration of 10 mg alemtuzumab (anti-CD52). Exacerbation of skin GvHD was documented in September 2013, followed by reinforced immune suppression with systemic steroids and extracorporeal photopheresis for 6 months followed by treatment with the Janus kinase (JAK) inhibitor ruxolitinib. At the regular visit 2 years after transplantation in September 2014, chronic GvHD was overall scored moderate with stomatitis (stage 1), and skin GvHD (stage 2). GvHD-targeting immune-suppressive treatment could eventually be stopped. At the time of writing, only very mild chronic GvHD (skin stage 1, oral stage 1) was documented. Since aSCT, chimerism analysis in myeloid cells and lymphocytes has been repeatedly performed as part of the routine clinical follow-up, always demonstrating 100% donor chimerism (last analysis in 2019). In November 2023, we further sorted CD4^+^ vs. CD8^+^ T cells vs. NK cells vs. monocytes of the patient and demonstrated 100% donor chimerism in all these immune cell subpopulations using a highly sensitive droplet-based PCR analysis (data not shown, see Methods).

Five years after aSCT, the patient presented with watery diarrhea. After stool multiplex-PCR ruled out bacterial, viral, or parasite pathogens, colonoscopy revealed a moderate left-sided colitis, histologically associated with non-specific inflammation but neither evidence of cytomegalovirus (CMV) infection nor GvHD (Lerner grade 0). Broad-spectrum antibiotics and short-term budesonide were associated with transient improvement. However, within a few weeks, the patient developed bloody diarrhea and abdominal cramps. Blood tests revealed leukocytosis, markedly elevated C-reactive protein, and a newly developed iron deficiency anemia. Stool calprotectin was > 800 ug/g stool, pathogen multiplex stool-PCR screening, including *Clostridioides difficile*, was negative, and the computer tomography (CT)-imaging demonstrated a severe pancolitis. A repeat colonoscopy confirmed an erosive pancolitis and a moderate terminal ileitis (Supplemental Fig. 1). Histologically, multiple crypt abscesses, distortion of the crypt architecture on colonic biopsies, and an erosive inflammation with cryptitis in the ileum were consistent with diagnosis of Crohn’s disease (CD) (Supplemental Fig. 1). No evidence of GvHD or CMV infection was found. Gastro-duodenoscopy performed at this time showed the absence of duodenal inflammation and no evidence of Whipple’s disease. High-dose prednisolone therapy with a tapering scheme, along with an infliximab induction therapy (5 mg/kg body weight), was initiated. The patient achieved remission which was maintained clinically, biochemically, and endoscopically up to the present with infliximab every 6–8 weeks in the absence of steroids. In this stable remission, the distribution of lymphocyte and T cell subpopulations in peripheral blood was analyzed, demonstrating normal T cell numbers with a preserved CD4^+^/CD8^+^ T cell ratio > 1. CD25^hi^CD127^lo^ regulatory CD4 T cell frequencies were also normal, naïve CD4 T cells were slightly low, and follicular T helper cells were relatively elevated compared to age- and sex-matched reference values (Table 1).

**Fig. 1:**
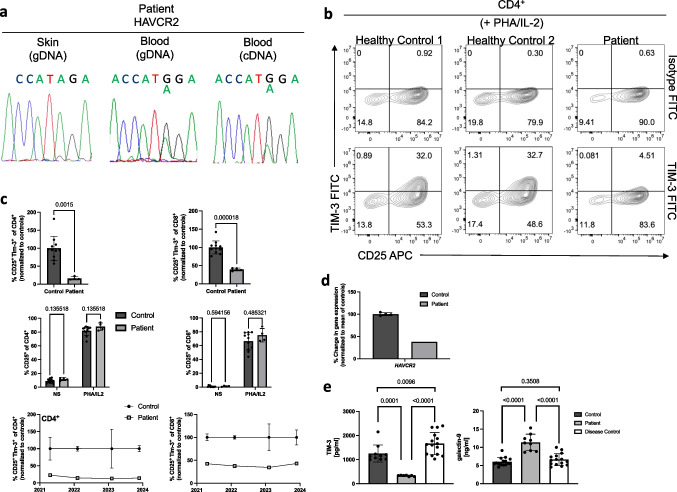
**a** Genomic DNA derived from skin or peripheral blood-derived PBMC of the index patient was generated, and a PCR product comprising the c.A291G *HAVCR2* variant was amplified. Sanger sequencing was performed, and chromatograms are depicted. **b**, **c** PBMCs of the index patient vs. healthy controls were stimulated with PHA/IL2 for 48 h, and TIM-3 vs. CD25 expression on CD4^+^ vs. CD8^+^ T cells was measured by flow cytometry. Representative flow cytometry plots are depicted in (**b**). Results from four independent experiments over the course of 2.5 years with a total of 11 healthy controls are summarized in (**c**). TIM-3 expression was normalized for each individual experiment to the mean of TIM-3 levels measured in parallel in healthy controls. **d** *HAVCR2* mRNA expression levels were quantified in patient-derived PBMCs by real-time PCR normalized to the mean of *HAVCR2* levels measured in healthy controls. **e** Patient serum was collected over the time course of 6 years (2017–2023), and levels of TIM-3 and Galectin-9 were quantified by ELISA and compared to control sera of healthy controls (control) and randomly assigned patients enrolled into our immune-dysregulation cohort (disease control). **c**, **e** Mean and standard deviation (SD) are depicted, and *p*-values were calculated using an unpaired *t*-test

Since the development of CD after aSCT is a very rare observation at our center and sparsely reported in the literature, we prepared genomic DNA from the patient’s blood and skin to search for rare immune gene variants linked to inborn errors of immunity (IEI)—a group of genetically determined immune system diseases often manifesting with gastrointestinal inflammation. Whole exome and Sanger sequencing of the blood-derived DNA detected a rare missense variant (c.A291G; p.I97M; rs35960726; allele frequency based on the gnomAD database: 0.002885) in the *HAVCR2* gene, which encodes for the immune-regulatory protein TIM-3 (Fig. 1a). In contrast to TIM-3 insufficiency associated with germline *HAVCR2* mutations [19, 20], our patient did carry the mutation specifically in hematopoietic cells as it was absent in skin-derived DNA (Fig. 1a). Whole exome sequencing data did not reveal additional disease-causing mutations in genes linked to IEI as listed in the most recent report by the International Union of Immunology Societies expert group [1]. We, however, cannot exclude that additional rare variants in IEI genes may have contributed to the clinical phenotype [21].

**Fig. 2:**
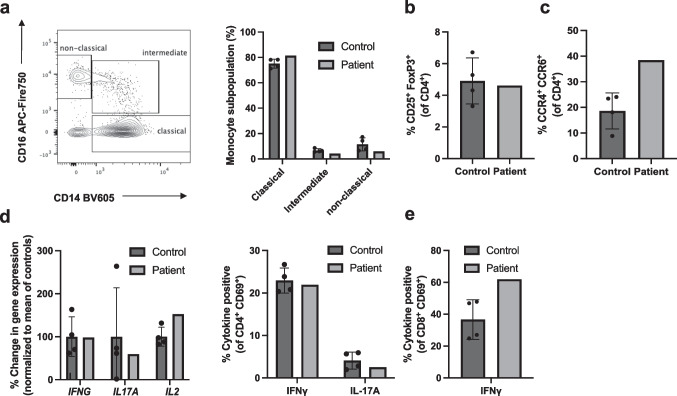
**a** Peripheral blood-derived monocytes of the patient *vs.* controls were phenotyped by flow cytometry. **b**, **c** The indicated T cell subsets of the patient vs. healthy controls were determined by flow cytometry. **d**, **e** PBMCs of the index patient *vs.* four controls were stimulated with agonistic anti-CD3 (clone: OKT3) and anti-CD28 (clone: CD28.2) for 4 h. mRNA expression levels of the indicated cytokines were quantified by real-time PCR (**d**) or the indicated cytokine expression assessed by intra-cellular staining and flow cytometry (**e**)

To measure whether the heterozygous *HAVCR2* mutation leads to reduced TIM-3 expression in immune cells, we in vitro stimulated patient-derived T cells with PHA/IL-2 for 48 h and measured TIM-3 expression by flow cytometry. Analysis of TIM-3 expression in stimulated T cells from a total of eleven unrelated healthy volunteers served as a control. Patient-derived T cells upregulated CD25 normally, while TIM-3 expression was approximately 20% of normal in CD4^+^ T cells (Fig. 1b, c). Reduced TIM-3 expression in the patient was a stable trait as it was measured on four occasions over a time span of more than 2.5 years (Fig. 1c). *HAVCR2* mRNA levels in non-stimulated PBMCs of the patient were lower compared to controls (Fig. 1d), arguing for enhanced RNA decay of the mutated *HAVCR2* allele. Stably reduced soluble TIM-3 levels [22] (sTIM-3) were measured by ELISA in the serum of the index patient using sera over a time course of 6 years (Fig. 1e). This was contrasted by elevated Galectin-9 levels in the patient’s serum, suggesting a negative feedback loop (Fig. 1e) similar to what has been described for other immune-regulating signaling pathways [23]. As myeloid cells have been demonstrated to be functionally shaped by TIM-3 [24], we additionally flow cytometrically phenotyped peripheral monocytes of the patient without detecting abnormal subset distribution (Fig. 2a). We further assessed patient-derived T cells ex vivo and following in vitro stimulation but did not find differences vs. controls with regards to expression of FOXP3 (Fig. 2b) or the levels of IFN-γ, IL-17A, and IL-2 (Fig. 2d). Notably, CCR6^+^CCR4^+^ T cells, which are linked to the Th17 lineage, were higher in the patient than observed in controls (Fig. 2c).

**Fig. 3:**
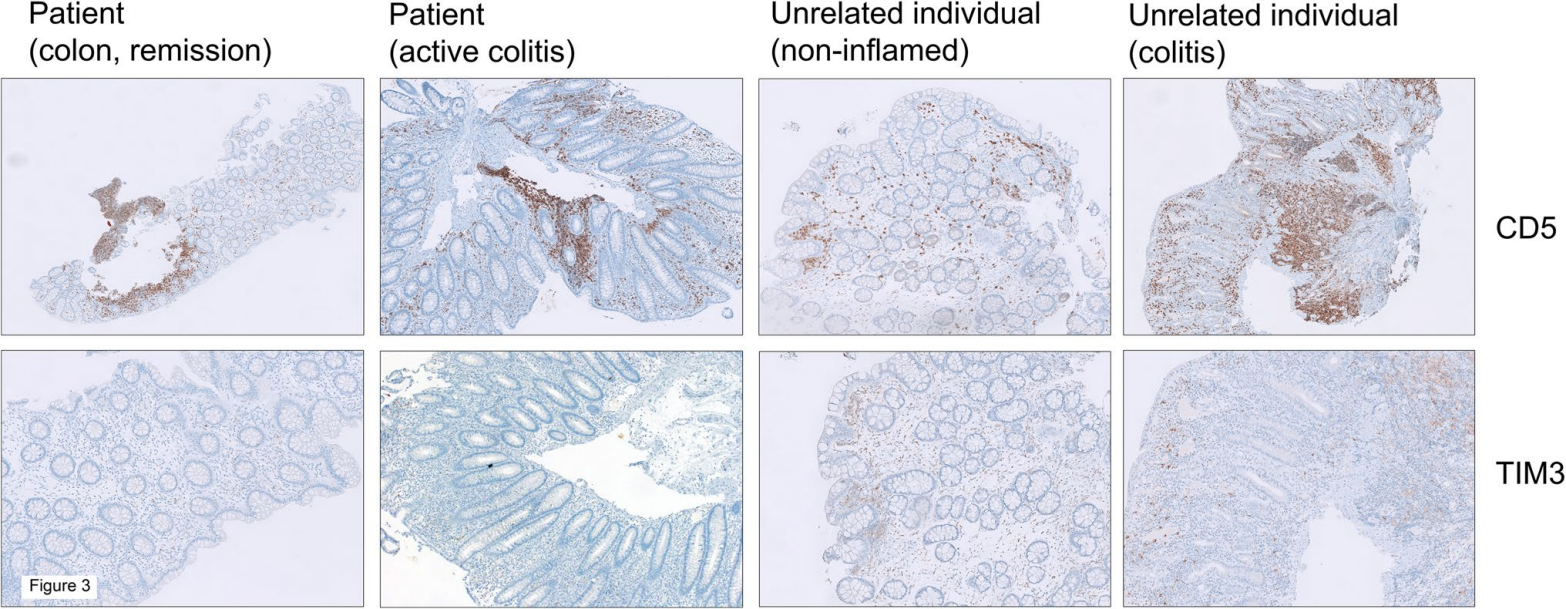
Colon sections of the index patient in active IBD *vs.* clinical remission (under infliximab therapy) were stained by immune histology for CD5 (a pan T cell marker) and for TIM-3 expression. Analysis of sections from non-inflamed *vs.* inflamed (IBD) colon tissue from non-related patients served as a control

The immune histology of patient-derived intestinal biopsies was performed to examine TIM-3 expression in T cells within intestinal tissue. While CD5^+^ T cells were easily detectable in the patient’s stomach, duodenum, ileum, and colon, TIM-3 positive lymphocytes were almost completely absent, independent of the IBD activity (Fig. 3, Supplemental Fig. 2). In contrast, TIM-3 positive lymphocytes were present in both inflamed and non-inflamed colon from unrelated individuals (Fig. 3). Control stainings revealed the presence of FOXP3 and ROR-γ positive cells predominantly in the inflamed colon of the patient and controls (Supplemental Figs. 3 and 4).

## Discussion

The c.A291G; p.I97M *HAVCR2*/TIM-3 mutation has been demonstrated to encode a loss of function allele and has, in homozygous but also in heterozygous states, been shown to cause subcutaneous panniculitis-like T cell lymphoma (SPTCL) and autoinflammatory disease [19]. Also, cases of autoinflammation in the absence of SPTCL have been recently linked to *HAVCR2* germline mutations [25, 26]. The I97M missense mutation causes TIM-3 misfolding, aggregation in the Golgi apparatus, and lack of surface expression [19]. In addition, our results suggest enhanced RNA decay of the mutated *HAVCR2* allele. It has been previously reported that heterozygous *HAVCR2* mutations, including the I97M found here, can be associated with TIM-3 insufficiency [19, 20]. In fact, a careful review of the immune-histological and clinical features of patients with heterozygous TIM-3 mutations did not reveal differences from patients with homozygous mutations [20]. The molecular reason for this is currently unknown but is supported by our own data showing TIM-3 expression around 20% of normal in CD4^+^ T cells associated with heterozygous carriage of the p.I97M missense mutation. Our patient’s *HAVCR2* mutation was donor-derived since donor chimerism was 100%. There was no overt immunologic disease reported in the stem cell donor. Potential stem cell donors undergo extensive health check-ups; however, there is currently no standard screening for IEI- or hematologic malignancy-related mutations [27, 28]. The clinical penetrance of disease associated with TIM-3 missense mutations, especially in heterozygous state, has, to our knowledge, not been studied. The clinical penetrance of IEI gene mutations in general is often rather low and is known to be influenced by environmental, genetic, and epigenetic factors [4]. TIM-3 expression is enhanced in ex vivo analyzed CD4^+^ T cells of the intestinal mucosa compared to the peripheral blood [29]. TIM-3 down-regulation has been described during human IBD flares [29, 30], and blockade of TIM-3 function exacerbates inflammation in murine colitis models [24, 31]. Vice versa, the TIM-3 ligand Galectin-9 has been recently demonstrated to reduce 2,4,6-trinitrobenzene sulfonic acid (TNBS)-induced colitis in mice [32]. Homozygous carriage of the TIM-3 Thr101Ile missense variant (rs147827860, not the same variant as detected in our patient) has been linked to human IBD [33]. These results are corroborated by recent genome-wide association studies demonstrating the linkage of TIM-3 mutations and CD [34]. Accordingly, insufficient expression of other regulatory immune checkpoint molecules, such as CTLA-4 [35, 36], or inactivation of their regulatory function by checkpoint inhibitors [37] can cause colitis. Thus, the link between TIM-3 deficiency and human IBD appears plausible.

## Conclusion

This is the first description of an acquired, hematopoietic cell-intrinsic, pathogenic *HAVCR2* mutation associated with TIM-3 deficiency in blood-derived and intestinal immune cells clinically linked with post-aSCT IBD.

### Supplementary Information

Below is the link to the electronic supplementary material.

Supplementary file1 (PDF 2747 KB) Supplemental Figure 1 Representative endoscopic appearance (top) and H+E conventional histology of colon tissue (bottom) of the index patient during active colitis are depicted.

Supplementary file2 (PDF 32934 KB) Supplemental Figure 2 Sections of the indicated intestinal tissues of the index patient in clinical remission (under infliximab therapy) were stained by immune-histology for T cells (CD5) and for TIM-3 expression.

Supplementary file3 (JPG 4707 KB) Supplemental Figure 3 Sections from the same tissue samples analyzed in Figure 2 were assessed by immune-histology for staining of T cells expressing the transcription factors Foxp3 and ROR-γt.

Supplementary file4 (JPG 4381 KB) Supplemental Figure 4 Sections of the indicated intestinal tissues of the index patient in clinical remission under infliximab therapy (same tissues as analyzed in Supplemental Figure 2) were analyzed by immune histology for Foxp3 and ROR-γt expressing T cells.

## Data Availability

The data underlying this article will be shared on reasonable request to the corresponding authors.
