# Supplementary figures and images for: Post-transplant Inflammatory Bowel Disease Associated with Donor-Derived TIM-3 Deficiency
Source: J Clin Immunol. 2024 Feb 16;44(3):63. doi: 10.1007/s10875-024-01667-z (PMC10873237; doi:10.1007/s10875-024-01667-z)

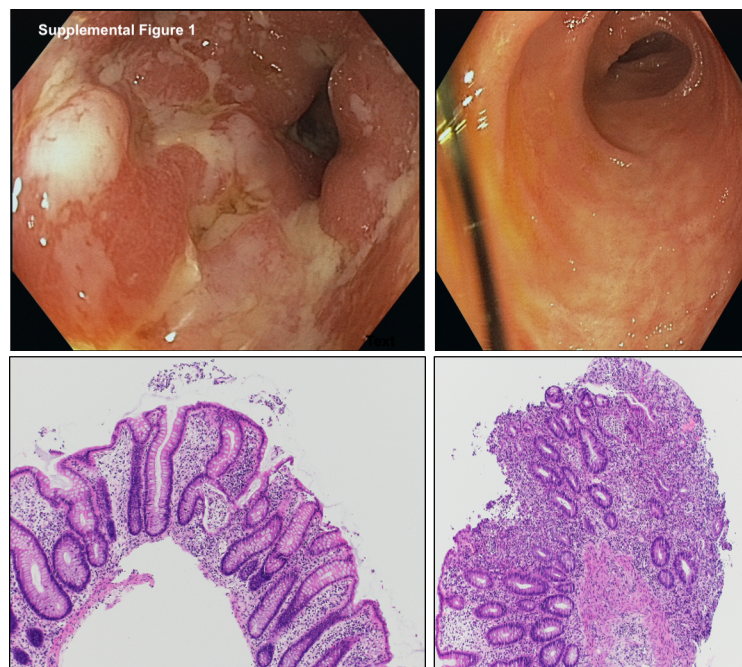

Supplement: Supplementary file 1 — Supplementary file1 (PDF 2747 KB) Supplemental Figure 1 Representative endoscopic appearance (top) and H+E conventional histology of colon tissue (bottom) of the index patient during active colitis are depicted. [file 10875_2024_1667_MOESM1_ESM.pdf]

Stomach

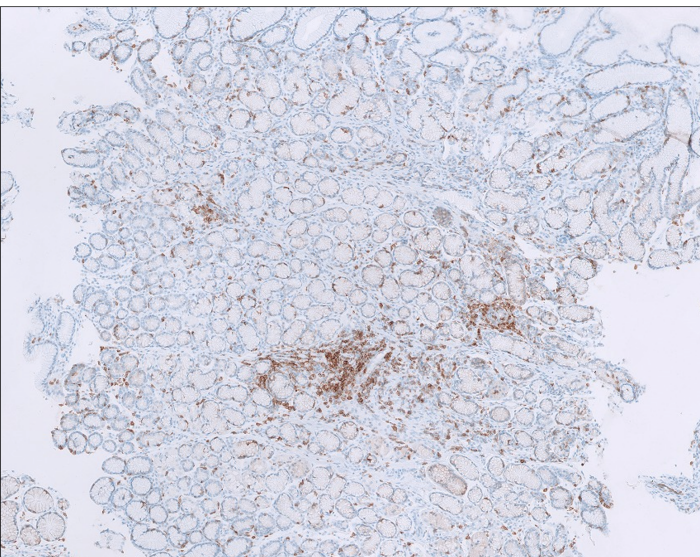

Duodenum

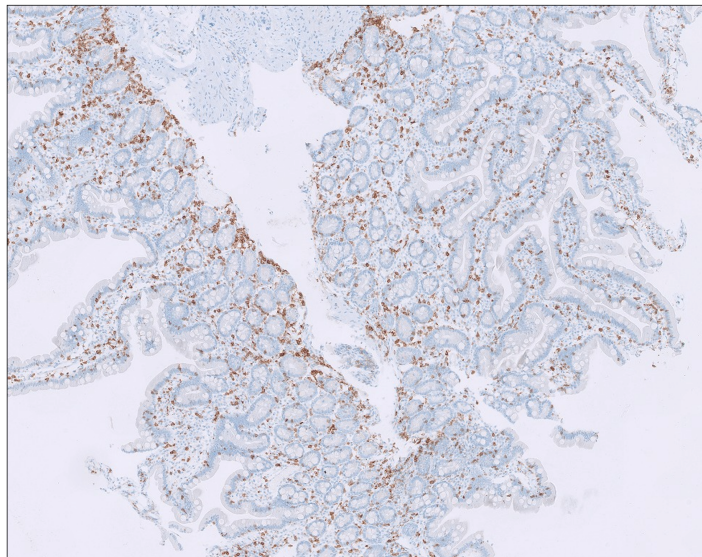

Terminal Ileum

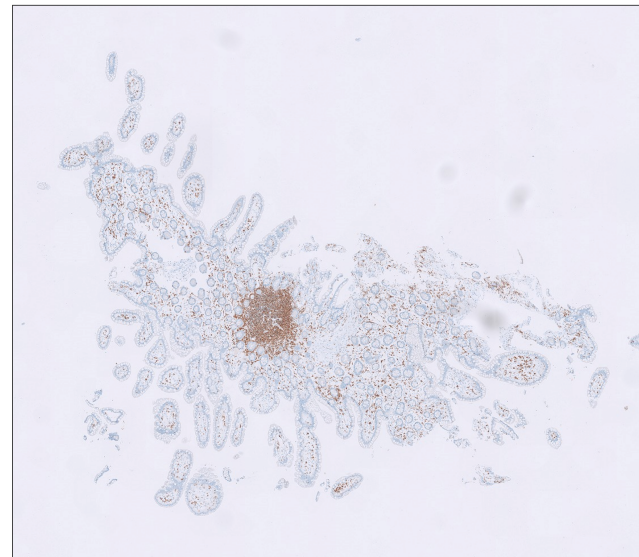

CD5

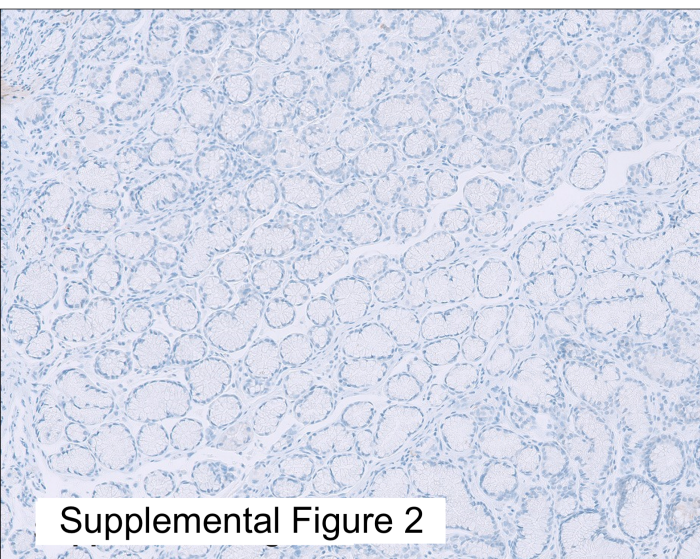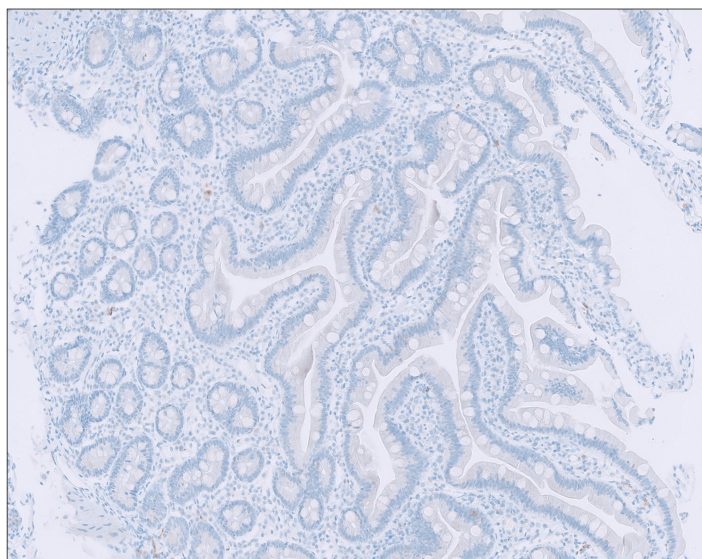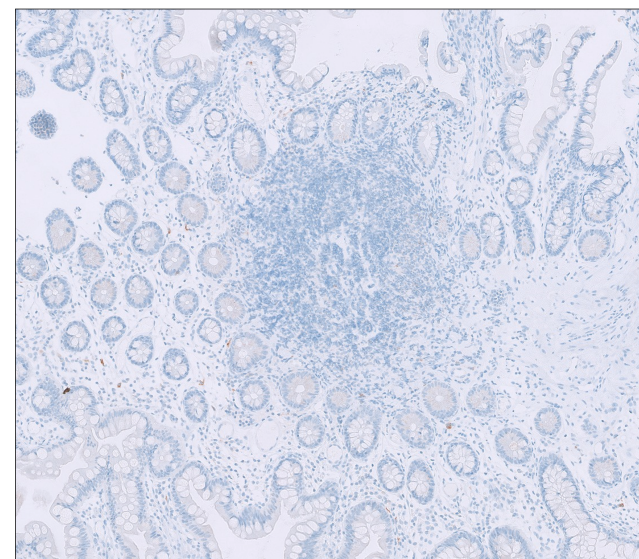

TIM3

Supplement: Supplementary file 2 — Supplementary file2 (PDF 32934 KB) Supplemental Figure 2 Sections of the indicated intestinal tissues of the index patient in clinical remission (under infliximab therapy) were stained by immune-histology for T cells (CD5) and for TIM-3 expression. [file 10875_2024_1667_MOESM2_ESM.pdf]

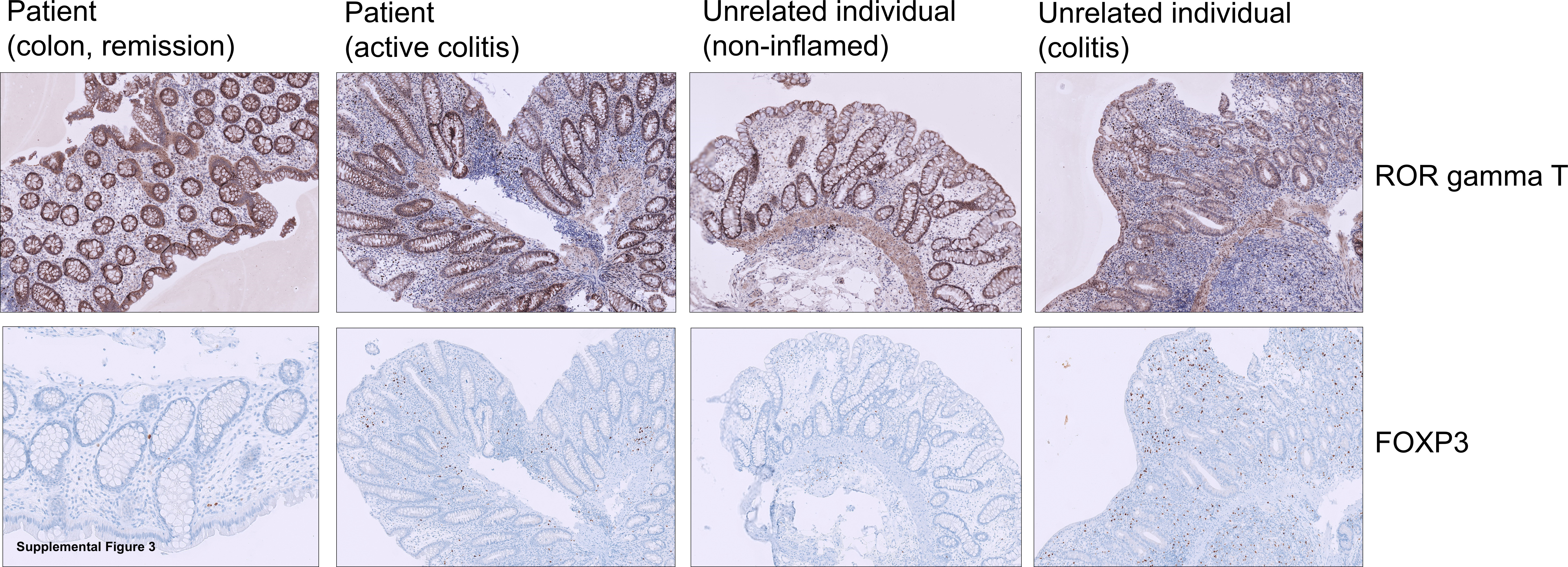

Supplement: Supplementary file 3 — Supplementary file3 (JPG 4707 KB) Supplemental Figure 3 Sections from the same tissue samples analyzed in Figure 2 were assessed by immune-histology for staining of T cells expressing the transcription factors Foxp3 and ROR-γt. [file 10875_2024_1667_MOESM3_ESM.jpg]

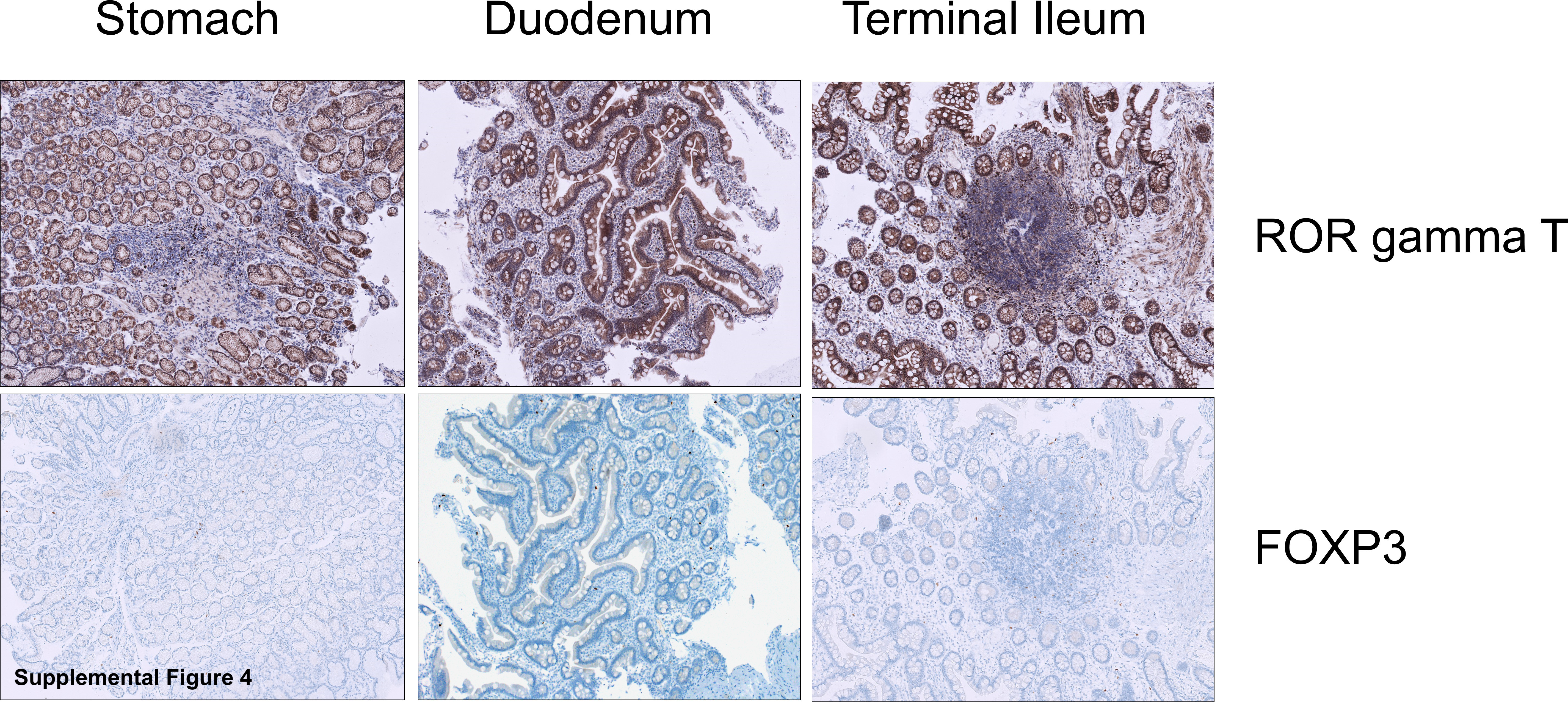

Supplement: Supplementary file 4 — Supplementary file4 (JPG 4381 KB) Supplemental Figure 4 Sections of the indicated intestinal tissues of the index patient in clinical remission under infliximab therapy (same tissues as analyzed in Supplemental Figure 2) were analyzed by immune histology for Foxp3 and ROR-γt expressing T cells. [file 10875_2024_1667_MOESM4_ESM.jpg]
